# Association of In Vivo Kidney Stiffness Measured by Multifrequency MR Elastography and Long‐Term Renal Function in Transplant Recipients and Living Donors

**DOI:** 10.1002/jmri.29799

**Published:** 2025-04-17

**Authors:** Stephan Rodrigo Marticorena Garcia, Jonas Oppenheimer, Tom Meyer, Frank Friedersdorff, Ingolf Sack

**Affiliations:** ^1^ Department of Radiology Charité—Universitätsmedizin Berlin Berlin Germany; ^2^ Clinic of Urology Charité—Universitätsmedizin Berlin Berlin Germany

**Keywords:** allograft function, biomechanical tissue properties, donor‐recipient pairs, kidney transplantation, multifrequency MR elastography, water diffusion

## Abstract

**Background:**

Kidney transplant (KTx) function assessment is important in treatment planning, while conventional MRI markers lack sensitivity for KTx function. Mechanical kidney properties may serve as MRI markers for renal allograft function.

**Hypothesis:**

To determine if multifrequency MR elastography (MRE) is associated with KTx function.

**Study Type:**

Prospective, longitudinal.

**Subjects:**

Twelve kidney donors (51 ± 9 years, 8 females) and 12 allograft recipients (48 ± 17 years, 2 females).

**Fieldstrength/Sequence:**

1.5 T, MRE, and diffusion‐weighted MRI based on spin‐echo echo‐planar imaging and T2‐weighted MRI volumetry.

**Assessment:**

Kidney donors were imaged pre‐ and post‐KTx, while allograft recipients were imaged post‐KTx. Renal function was assessed using creatinine levels (at 1 week, 1 month, and 3 years post‐KTx in recipients) and glomerular filtration rate (GFR; donors pre‐KTx) based on Tc‐99m‐MAG3 scintigraphy. Kidney volumes were measured by MRI segmentations, and ADC, shear‐wave speed (SWS), and loss‐angle maps were reconstructed. The correlations between MR parameters, GFR, and creatinine level (minimum value over study period) were investigated.

**Statistical Tests:**

Wilcoxon tests and Pearson correlation coefficients (*R*). A *p* < 0.05 was considered statistically significant.

**Results:**

No significant lateral differences in renal volume (153 ± 34 cm^3^, *p* = 0.34) or SWS (2.5 ± 0.4 m/s, *p* = 0.20) were found pre‐KTx. Volume and SWS increased significantly post‐KTx in reference kidneys (volume: +13%, SWS: +11%) and in transplants in recipients (volume: +20%, SWS: +32%). SWS, but not volume (*p* = 0.75), correlated positively with GFR in donors pre‐KTx (*R* = 0.67) while both SWS (*R* = ‑0.62) and volume (*R* = −0.77) negatively correlated with creatinine levels post‐KTx. ADC was sensitive to KTx‐associated changes in renal function in donors (3% ± 5%) but not recipients (*p* = 0.88).

**Data Conclusion:**

MRE provides valuable information on renal function and could serve as a baseline for longitudinal monitoring of kidney transplants. Parenchymal stiffening post‐KTx had significantly larger effect sizes in denervated allografts than in reference donor kidneys with intact autoregulation of renal blood flow.

**Evidence Level:**

2.

**Technical Efficacy:**

Stage 2.

1

**Plain Language Summary**

- Because existing MRI markers lack accuracy, this study sought a more sensitive and noninvasive way to assess kidney transplant function.
- Therefore, the authors used multifrequency MR elastography (MRE) to measure renal stiffness in 12 donors and 12 recipients and compared biomechanical tissue properties with kidney function using glomerular filtration rate and creatinine levels over a 3‐year observation period.
- The results showed that renal stiffness increased after transplantation and that higher stiffness was associated with better renal function.
- MRE outperformed traditional MRI measures such as MRI volumetry and water diffusion in measuring renal function.

## 2 Introduction

The kidneys play a central role in removing waste products from the bloodstream and excreting urine. Chronic kidney disease (CKD) typically develops gradually, often without symptoms [1]. As it progresses, CKD becomes increasingly debilitating, with limited opportunity for reversal at later stages. CKD affects nearly 100 million Europeans, with another 300 million at risk, and is expected to become the fifth leading cause of death worldwide by 2040 [2]. In the most advanced stages, patients need kidney replacement therapy such as dialysis or kidney transplantation (KTx) to survive and maintain quality of life [3]. Unfortunately, compatible donor kidneys are not available for all patients with kidney failure eligible for transplantation [4]. Receiving a kidney from a living donor has many advantages over deceased kidney donation. In general, a kidney from a living donor lasts longer and the waiting period is shorter [5]. However, finding a suitable donor for kidney replacement therapy is a daunting and intense process, particularly as most donors are healthy individuals with little preceding contact with the health care system [6]. Renal scintigraphy is the preoperative reference standard for the selection of a kidney to be harvested for transplantation [7].

Over the past few decades, MRI has advanced the process of finding suitable living kidney donors [8, 9, 10]. Nonetheless, challenges continue to exist relating to the subjective interpretation of complex MRI data, as identifying the ideal donor requires a nuanced understanding of various anatomical and physiological factors. Therefore, MRI in KTx requires a multidisciplinary approach involving trained radiologists, nephrologists, and transplant surgeons. Quantitative MRI methods such as relaxation time mapping [11], diffusion‐weighted imaging (DWI) [12, 13], perfusion MRI [14], and MRI volumetry [15] have the potential to facilitate an objective matching of suitable donor‐recipient pairs for KTx.

A fundamental biophysical tissue property that can be measured by MRI is viscoelasticity [16]. Renal stiffness (storage modulus) and viscosity (loss modulus) can be quantified by magnetic resonance elastography (MRE) with high spatial resolution and interindividual consistency [17, 18]. Recently, multifrequency MRE with tomoelastography postprocessing [19] has demonstrated high sensitivity for assessing renal perfusion and function in lupus nephritis [20] and IgA nephropathy [21]. In renal allografts, higher stiffness values in MRE provide information on allograft function [22] and the success of antiviral therapy against hepatitis C virus [23]. In a CKD rat model, increased renal stiffness has been associated with adenine‐induced tubular dilation and increased intraluminal pressure [24]. These in vivo results are supported by MRE in ex vivo kidneys, where measured renal stiffness has been found to correlate linearly with perfusion pressure [25, 26].

However, classical single‐frequency MRE has yielded inconsistent results in the kidney, including lack of sensitivity [27] and poor diagnostic power in CKD [28], and softening [29] or stiffening [30, 31] with renal fibrosis, arguably indicating overlapping effects of matrix accumulation, turgor, and hemodynamic changes [32, 33, 34]. Despite extensive studies in healthy volunteers at various hydration levels [25, 35, 36], clinical data on how renal viscoelasticity is affected by changes in renal perfusion occurring in the setting of controlled interventions are scarce. Specifically, studies comparing kidney mechanical properties in living kidney donors before transplantation (pre‐KTx) and after transplantation (post‐KTx) in both donors and recipients are lacking.

Thus the aim of this study was to investigate the use of multifrequency MRE to noninvasively compare renal stiffness in KTx donors and recipients pre‐ and post‐KTx. A further aim was to determine the correlation of stiffness with clinical function markers such as creatinine levels, glomerular filtration rate (GFR), and renal scintigraphy in order to determine the potential of MRE to provide quantitative MRI markers for the assessment of kidney structure and function in the setting of living kidney donation.

## 3 Methods

This prospective study complied with the Declaration of Helsinki and was approved by the internal review board of Charité—Universitätsmedizin Berlin (EA1/144/20). All participants gave written informed consent.

### 3.1 Study Population

**FIGURE 1:**
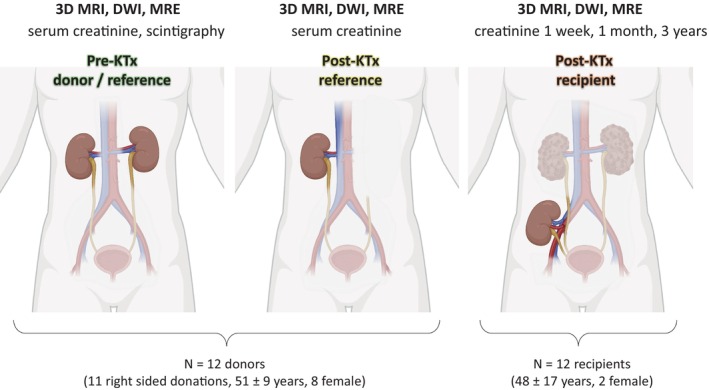
Study design with examinations performed, participants, and anatomical reference.

In this study, 24 study participants (12 kidney donors, 51 ± 9 years, 8 females; 12 kidney recipients, 48 ± 17 years, 2 females) underwent multiparametric MRI including 3D T2‐weighted MRI for volume quantification, diffusion‐weighted MRI (DWI) for quantification of the apparent diffusion coefficient (ADC), and multifrequency MRE for quantification of viscoelastic tissue properties. This MRI protocol was implemented twice in donors 1 ± 0 days pre‐KTx and 11 ± 4 days post‐KTx and once in recipients, 19 ± 6 days post‐KTx. All donors underwent clinical function tests including blood markers for creatinine before and after KTx on the same day as MRI. In addition, renal Tc‐99 m‐mercaptoacetyltriglycine (MAG3) scintigraphy was performed as a GFR‐equivalent [37] in all donors within 2 weeks before the first MRI. In recipients, creatinine levels were monitored at 1 week, 1 month, and 3 years after transplant. MRI‐creatinine correlations were based on the best function value, that is, the minimum creatinine level over 3 years. The study protocol is summarized in Figure 1.

### 3.2 Imaging Protocol

All quantitative MRI examinations were performed on a 1.5‐T MRI system (Magnetom Sonata, Siemens, Erlangen, Germany) equipped with a 12‐channel phased‐array surface coil. Images were obtained in para‐coronal slice orientation covering both kidneys in donors and the allograft kidney in recipients. DWI was performed with a 2D spin‐echo echo‐planar imaging (EPI) sequence 128 × 128 × 11 matrix size and 2.7 × 2.7 × 5 mm^3^ acquired voxel size. *b* values of 0 and 500 s/mm^2^ were acquired to compute ADC maps [21]. MRI volumetry was based on 3D T2‐weighted MRI using a half‐Fourier single‐shot turbo spin‐echo (HASTE) sequence with 192 × 256 × 23 matrix size and 1.6 × 1.6 × 7 mm^3^ reconstructed voxel size, taking into account that slice thickness does not significantly affect volume measures [38]. Kidney volumes were measured by manual segmentation in three dimensions. Multifrequency MRE was performed using a spin‐echo EPI sequence [39] and external vibration frequencies of 40, 50, 60, and 70 Hz as detailed in [21]. In brief, eight wave phase offsets were recorded for each of the three motion directions. The imaging volume consisted of 11 contiguous paracoronal slices with a field of view of 260 × 260 mm^2^ (matrix size, 104 × 104) and 2.5 × 2.5 × 2.5 mm^3^ acquired voxel size. To increase the signal‐to‐noise ratio, the signal was averaged over two repetitions. Further imaging parameters were as follows: repetition time, 1200 ms; echo time, 55 ms; parallel imaging with a GRAPPA factor of 2; motion encoding gradient (MEG) frequency, 48.45 Hz for mechanical frequencies of 40, 50, and 60 Hz, and 52.41 Hz for mechanical frequency of 70 Hz; and MEG amplitude of 25 mT/m. The full set of multifrequency MRE data was acquired within 4 min with subjects breathing freely. Vibrations were induced by four compressed air drivers fixed to the body surface with Velcro strips. In kidney donors, the drivers were symmetrically placed in anterior and posterior positions at the kidney level. In recipients, one anterior driver was placed directly above the KTx while two air drivers were placed in posterior position at the same level as the anterior driver. MRE postprocessing was performed using a server‐based software (https://bioqic‐apps.charite.de) [19] which provides implementations of wavenumber‐based (k‐) multifrequency elasto‐visco inversion (k‐MDEV) for reconstruction of shear‐wave speed (SWS) [40] and MDEV for reconstruction of the loss angle, 𝜑, of the complex shear modulus [41]. SWS (in m/s) was determined as a surrogate marker of stiffness while 𝜑 (in rad) was used as a viscosity‐related parameter that measures tissue fluidity [42] The fluidity of solid tissue is related to the slope of SWS dispersion over frequency [17].

Regions of interest (ROIs) were manually drawn by a resident radiologist with > 1 year clinical experience in MRE for the entire renal parenchyma excluding the renal pelvis. The same kidney was analyzed in the donor before transplantation and then in the recipient after transplantation. The untransplanted kidney in the donor subject is referred to as the reference.

### 3.3 Statistical Analysis

Pre‐ and postoperative parameters were compared in the reference kidneys in the donors (pre‐KTx reference, post‐KTx reference) and between donor‐recipient pairs (pre‐KTx donor, post‐KTx recipient) using paired nonparametric tests (Wilcoxon signed rank test). The relationship between MRI parameters (SWS, 𝜑, VOL, ADC) and clinical parameters was assessed using Pearson's linear correlation coefficient, R assuming normal distribution for all pooled group data. A *p* < 0.05 was considered significant.

## 4 Results

**FIGURE 2:**
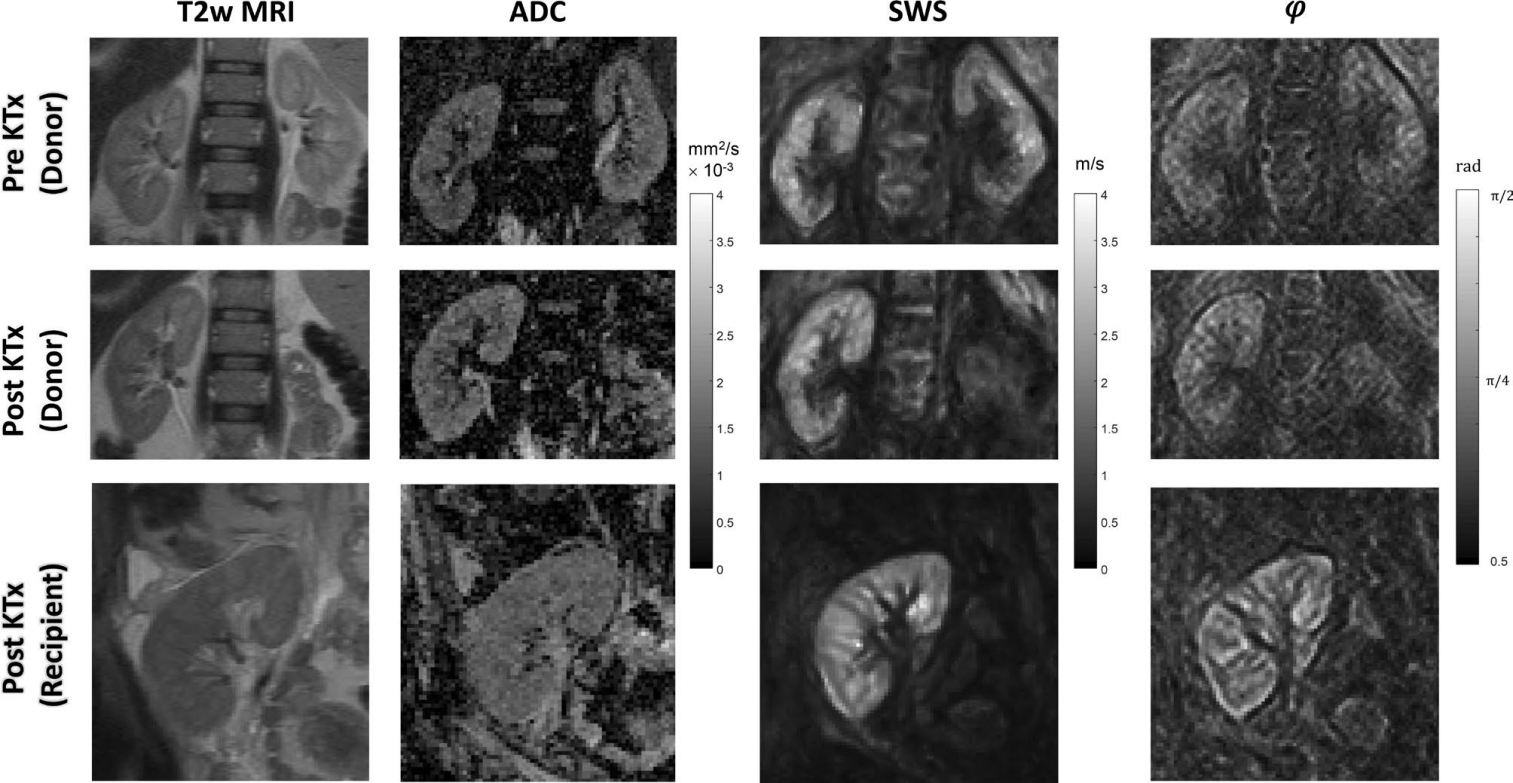
Representative images obtained in a 50‐year‐old male kidney donor and a 46‐year‐old female recipient. Shown are T2‐weighted MRI (T2w MRI), diffusion‐weighted MRI providing the apparent diffusion coefficient (ADC), and MRE providing shear wave speed (SWS) and loss angle (φ) as surrogate markers of tissue stiffness and tissue fluidity in a coronal plane.

**FIGURE 3:**
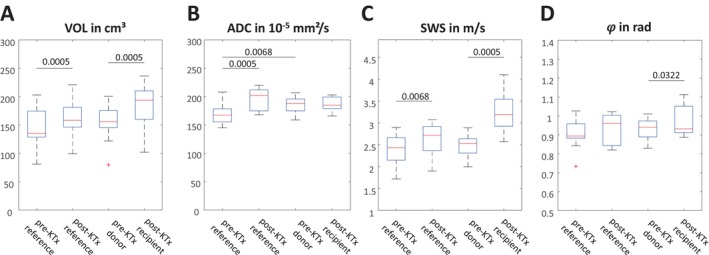
Group statistical plots of volume (A, VOL), water diffusion (B, ADC), tissue stiffness (C, SWS) and tissue fluidity (D, loss angle 𝜑) for reference kidneys remaining in donors before (pre‐KTx reference) and after (post‐KTx reference) transplantation as well as for allografts before (pre‐KTx donor) and after (post‐KTx recipient) transplantation. *p* values are provided above the horizontal lines.

**FIGURE 4:**
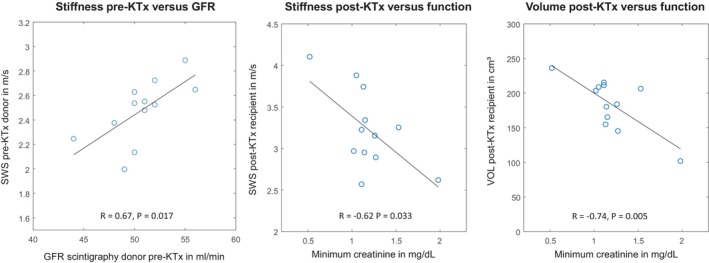
Correlation analysis for stiffness and volume versus renal function measurement based on renal scintigraphy (depicting GFR in mL/min) and creatinine level (minimum creatinine value over a follow‐up period of 3 years in mL/dL).

Eleven of the 12 donated kidneys were from the left side and none were rejected after transplantation. Mean ischemia time was 218 ± 66 min. MRI volumetry, DWI, and MRE were successfully performed in all donor‐recipient pairs and yielded a total of four in vivo biophysical parameters related to tissue volume, water diffusion, stiffness, and tissue fluidity (VOL, ADC, SWS, and 𝜑) over three scans and at different time points (pre‐KTx reference [Scan 1], post‐KTx reference [Scan 2], pre‐KTx donor [Scan 1], post‐KTx recipient [Scan 3]). Figure 2 shows representative images of T2 weighted‐MRI, ADC, and MRE for a donor‐recipient pair before and after KTx. Group statistical values are compiled in Figure 3. In donor kidneys pre‐KTx, no significant lateral differences were found for SWS (*p* = 0.34), VOL (*p* = 0.20), or 𝜑 (*p* = 0.69). Hence, averaged values for left and right kidneys were subsequently analyzed, yielding a mean ± standard deviation (SD) of SWS = 2.5 ± 0.4 m/s, 𝜑 = 0.92 ± 0.08 rad, and VOL = 153 ± 34 cm^3^. Pre‐KTx ADC was significantly higher (3% ± 5%) in the donated kidney (186 ± 14 × 10^−5^ mm^2^/s) than in the reference kidney (169 ± 19 × 10^−5^ mm^2^/s, Figure 3). Compared with pre‐KTx reference values, SWS, VOL, and ADC were significantly increased in the reference kidney post‐KTx, by 11% ± 11%, 13% ± 7%, 17% ± 15% to 2.5 ± 0.4 m/s, 153 ± 34 cm^3^, and 182 ± 23 × 10^−5^ mm^2^/s, respectively, whereas 𝜑 did not change significantly (*p* = 0.21). ADC did not change significantly after implantation of donor allografts into recipients (*p* = 0.88), while SWS, 𝜑, and VOL increased to 3.2 ± 0.5 m/s (32% ± 25%), 0.97 ± 0.08 rad (2% ± 6%), and 184 ± 37 cm^3^ (20% ± 9%), respectively, indicating expansion, stiffening, and a shift toward fluid‐like properties of allograft kidneys after denervation and re‐exposure to the bloodstream following non‐physiological anastomosis. Post‐KTx recipient values were negatively correlated with the minimum serological creatinine value over the period from 1 week to 3 years with *R* = −0.62 for SWS and *R* = −0.74 for VOL (Figure 4). Only SWS in the pre‐KTx donor kidney was associated with GFR assessed with renal scintigraphy, having a positive correlation of *R* = 0.67. All group values are summarize in Table 1.

**TABLE 1:** Group values (mean, SD) of quantitative MRI and renal function ****markers.

	SWS (m/s)	Loss angle (𝜑) (rad)	Volume (cm^3^)	ADC (10^−5^ mm^2^/s)	Scintigraphy (mL/min/1.73 m^2^)	Minimum creatinine (mg/dL)
Pre‐KTx reference	2.39 ± 0.35	0.91 ± 0.08	144.7 ± 32.2	168.8 ± 17.8	49.3 ± 3.1	—
Post‐KTx reference	2.63 ± 0.34**	0.93 ± 0.08	161.8 ± 31.7***	196.0 ± 18.7***	—	1.08 ± 0.19
Pre‐KTX donor	2.48 ± 0.24	0.93 ± 0.05	153.9 ± 29.9	185.6 ± 13.6**	50.7 ± 3.1	—
Post‐KTx recipient	3.23 ± 0.46+++	0.97 ± 0.08+	184.5 ± 35.9+++	187.3 ± 11.8	—	1.19 ± 0.33

*Note*: Scintigraphy = side‐separated Tc‐99 m‐mercaptoacetyltriglycine (MAG3) scintigraphy—glomerular filtration rate equivalent.

*p < 0.05, **p < 0.01, ***p < 0.001 (relative to the pre‐KTx reference kidney).

+*p* < 0.05, ++*p* < 0.01, +++*p* < 0.001 (relative to the pre‐KTx donor kidney).

## 5 Discussion

In this study, in vivo changes in kidney viscoelasticity after renal allograft transplantation were quantified both in donors and recipients. The results showed that stiffness and loss‐angle‐related tissue fluidity were significantly higher in the transplanted kidney in the recipient (post‐KTx recipient) than in the same kidney in the pre‐KTx donor. Thus, upon surgical anastomosis, this study showed that renal allografts expand, stiffen, and show higher tissue fluidity, consistent with previous work in an ex vivo model of perfused kidney transplants [26]. Changes in renal allograft perfusion result from the unphysiological anastomosis of the renal artery and vein to the main iliac vessels [43]. In addition, transection of renal nerves, which are important for the autoregulation of renal perfusion, results in unregulated blood flow into the kidney and a subsequent vascular turgor, which may account for the observed increase in effective stiffness properties beyond normal values [22]. In addition to these hemodynamic effects, greater metabolic activity after functional adaptation could have contributed to the observed increases in volume and stiffness in allografts [44] and in reference kidneys remaining in donors. Since perfusion regulation remains intact in the reference kidney, the increase in total kidney volume observed in this study after KTx might be attributable to cellular hypertrophy due to increased metabolism, consistent with the observations of Halleck et al. in allografts [44]. This is supported by the increases in ADC values found in the reference kidneys after KTx and by similar findings in 13 KTx donor‐recipient pairs investigated in a previous study with a similar design [45]. While higher water diffusivity due to cell hypertrophy has been reported for the liver [46, 47], no such effect occurred in donated kidneys in the current study, consistent with another previous study [45]. Furthermore, renal blood oxygenation level‐dependent imaging has shown that reference kidneys after KTx have higher oxygen levels, possibly due to greater metabolic activity, while transplanted kidneys show no such change, similar to the ADC value results in the current study [48]. The direct link between metabolism and in vivo organ stiffness has recently been demonstrated in rabbit hepatocytes [49]. In rats, pregnancy‐induced cell hypertrophy has been shown to lead to higher liver stiffness, presumably due to turgor, which is translated into coarse‐grained organ stiffness by hyperelastic stiffening of cell membranes [47]. These examples of MRE sensitivity to cell swelling and metabolism, together with a positive correlation between renal perfusion and stiffness [25, 26, 50], may explain the sensitivity of tissue stiffness to renal function markers that has been observed in the current study.

A further indication of the potential link between perfusion and tissue mechanics is the correlation observed between SWS pre‐KTx in donors and GFR‐scintigraphy. MRE could provide supplementary information on perfusion and fibrosis and could additionally serve as a baseline for longitudinal monitoring. Moreover, the negative correlation of SWS post‐KTx in recipients with creatinine level suggests that allograft stiffness is associated with long‐term renal function. It should be noted that diurnal changes in blood markers such as creatinine cause variability that may confound the assessment of correlation with inherent tissue markers such as stiffness. In the current study, to minimize outliers and put statistical weight onto maximum functional performance, MRI‐creatinine correlations were based on the best function value, that is, the minimum creatinine value over 3 years. Unlike creatinine, cold ischemia time did not correlate with MRI findings. It may be that ischemia times in the study population were too short to induce structural renal parenchymal damage severe enough to be detectable by MRI parameters [51].

## 6 Limitations

First, there was a small number of donor‐recipient pairs. Therefore, our data do not allow the derivation of thresholds or the prediction of long‐term allograft function. Second, renal perfusion measurement by scintigraphy as a function marker could only be performed once in donors, before transplantation, due to clinical restrictions on radiation exposure. As blood markers undergo diurnal variation, it would be desirable to perform scintigraphy in recipients for direct assessment of renal function, which is precluded by current safety considerations. Furthermore, the time interval of MRI scanning post‐KTx was different among subjects, potentially leading to different levels of organ adaptation. However, with more than a week minimum scanning interval post‐KTx, our MRI parameters could be considered stable against transplant‐related physiological fluctuations such as renal perfusion. The fact that none of the grafts were rejected also supports the stability of our measured MRI parameters.

## 7 Conclusion

This study investigated the sensitivity of MRI‐derived biophysical imaging markers, viscoelasticity and water diffusion, to changes in structure and function occurring in human kidneys following transplantation. The study found a significant increase in tissue stiffness, viscosity‐related loss angle, and volume of the donor kidney after transplantation, while water diffusion did not change significantly. Similarly, kidneys remaining in living donors showed changes in stiffness, volume, and water diffusion. Stiffness was positively correlated with renal function as quantified by renal scintigraphy before transplantation and negatively correlated with minimum creatinine levels during long‐term follow‐up of recipients over 3 years. These results indicate that multifrequency MRE may facilitate decision‐making in living donor kidney transplantation.
